# FAST: FAST Analysis of Sequences Toolbox

**DOI:** 10.3389/fgene.2015.00172

**Published:** 2015-05-19

**Authors:** Travis J. Lawrence, Kyle T. Kauffman, Katherine C. H. Amrine, Dana L. Carper, Raymond S. Lee, Peter J. Becich, Claudia J. Canales, David H. Ardell

**Affiliations:** ^1^Quantitative and Systems Biology Program, University of California, MercedMerced, CA, USA; ^2^Molecular Cell Biology Unit, School of Natural Sciences, University of California, MercedMerced, CA, USA; ^3^Department of Viticulture and Enology, University of California, DavisDavis, CA, USA; ^4^School of Engineering, University of California, MercedMerced, CA, USA

**Keywords:** Unix philosophy, MultiFASTA, pipeline, bioinformatic workflow, open source, BioPerl, regular expression, NCBI taxonomy

## Abstract

FAST (FAST Analysis of Sequences Toolbox) provides simple, powerful open source command-line tools to filter, transform, annotate and analyze biological sequence data. Modeled after the GNU (GNU's Not Unix) Textutils such as grep, cut, and tr, FAST tools such as fasgrep, fascut, and fastr make it easy to rapidly prototype expressive bioinformatic workflows in a compact and generic command vocabulary. Compact combinatorial encoding of data workflows with FAST commands can simplify the documentation and reproducibility of bioinformatic protocols, supporting better transparency in biological data science. Interface self-consistency and conformity with conventions of GNU, Matlab, Perl, BioPerl, R, and GenBank help make FAST easy and rewarding to learn. FAST automates numerical, taxonomic, and text-based sorting, selection and transformation of sequence records and alignment sites based on content, index ranges, descriptive tags, annotated features, and in-line calculated analytics, including composition and codon usage. Automated content- and feature-based extraction of sites and support for molecular population genetic statistics make FAST useful for molecular evolutionary analysis. FAST is portable, easy to install and secure thanks to the relative maturity of its Perl and BioPerl foundations, with stable releases posted to CPAN. Development as well as a publicly accessible Cookbook and Wiki are available on the FAST GitHub repository at https://github.com/tlawrence3/FAST. The default data exchange format in FAST is Multi-FastA (specifically, a restriction of BioPerl FastA format). Sanger and Illumina 1.8+ FastQ formatted files are also supported. FAST makes it easier for non-programmer biologists to interactively investigate and control biological data at the speed of thought.

## 1. Introduction

Bioinformatic software for non-programmers is traditionally implemented for user convenience in monolithic applications with Graphical User Interfaces (GUIs) (Smith et al., 1994; Stothard, 2000; Rampp et al., 2006; Librado and Rozas, 2009; Waterhouse et al., 2009; Gouy et al., 2010). However, the monolithic application paradigm is easily outscaled by today's big biological data, particularly Next Generation Sequencing (NGS) data at gigabyte- and terabyte-scales. Better empowerment of non-programmers for genome-scale analytics of big biological data has been achieved through web-based genome browser interfaces (Markowitz et al., 2014; Cunningham et al., 2015; Rosenbloom et al., 2015). On the other hand, for smaller datasets, sequence and alignment editor applications encourage manual manipulation of data, which is error-prone and essentially irreproducible. To reduce error and increase reproducibility in the publishing of bioinformatic and biostatistical protocols it is important to facilitate the documentation and automation of data science workflows through scripts and literate programming facilities (Knuth, 1984) such as emacs org-mode (http://orgmode.org), as demonstrated in, for example (Delescluse et al., 2012) that both completely document and encode scientific workflows for machine processing of biological data.

Reproducibility in bioinformatics and biostatistics protocols is crucial to maintaining public trust in the value of its investments in high-throughput and high-dimensional measurements of complex biological systems (Baggerly and Coombes, 2009; Hutson, 2010; Baggerly and Coombes, 2011; Huang and Gottardo, 2013). In one analysis, only two of 18 published microarray gene-expression analyses were completely reproducible, in part because key analysis steps were made with proprietary closed-source software (Ioannidis et al., 2008). Furthermore, even though analytical errors are a major source of retractions in the scientific literature (Casadevall et al., 2014), peer-review and publication of scientific data processing protocols is generally not yet required to publish scientific studies. Adequate documentation of bioinformatic and biostatistical workflows and open source sharing of code upon publication (Peng, 2009) facilitates crowd-sourced verification, correction and extension of code-based analyses (Barnes, 2010; Morin et al., 2012), and reuse of software and data to enable more scientific discovery returns from public data (Peng, 2011). Peer review and publication of the data science protocols associated to scientific studies stems temptation to overinterpret results and encourages more objectivity in data science (Boulesteix, 2010). The ultimate remedy for these problems is to expand literacy in modern computational and statistical data science for science students in general (Morin et al., 2012; Joppa et al., 2013).

Web-based open-source workflow suites such as Galaxy (Blankenberg and Hillman-Jackson, 2014), Taverna (Oinn et al., 2006) and BioExtract (Lushbough et al., 2011) are a recent innovation in the direction of greater reproducibility in bioinformatics protocols for genome-scale analytics. However, the most powerful, transparent and customizable medium for reproducible bioinformatics work is only available to bioinformatics specialists and programmers through Application Programming Interfaces (APIs) such as BioPerl and Ensembl (Yates et al., 2015).

Yet workflow design suites and programming APIs require dedication and time to learn. There is a need for more bioinformatics software in between GUIs and APIs, that empowers non-programmer scientists and researchers to interactively and reproducibly control, process and analyze their data without manual interventions. Closer inspection of data and interactive construction and control of data workflows makes it so much easier to rapidly prototype error-free workflows, nipping errors in the bud that can completely confound downstream analyses. In scientific computing, the time-tested paradigm for rapid prototyping of reproducible data workflows is the Unix command-line.

In this tradition we here present FAST: FAST Analysis Sequences Toolbox, modeled after the standard Unix toolkit (Peek, 2001), now called Coreutils. The FAST tools follow the Unix philosophy to “do one thing and do it well” and “write programs to work together.” (Stutz, 2000). FAST workflows are completely automated; no manual interventions to data are required. FAST falls between a GUI and an API, because it is used through a Command-Line Interface (CLI). Although the FAST tools are written in Perl using BioPerl packages (Stajich et al., 2002), FAST users do not need to be able to program Perl or know BioPerl. FAST users only need basic competence in Unix and the modest skill to compose command pipelines in the Unix shell. FAST therefore supports an emerging movement to empower non-programmer biologists to learn Unix for scientific computing. Books and courses in this emerging market include the recent “UNIX and Perl to the Rescue!” (Bradnam and Korf, 2012) and the Software Carpentry and Data Carpentry Foundations workshops (Wilson, 2014).

Unix command pipe-lines are the paradigmatic example of the “pipes and filters” design pattern that embodies serial processing of data through sequences of modular and reuseable computations. The “pipes and filters” design pattern is a special case of component-based software engineering (McIlroy, 1969) and a core paradigm in software architecture (Garlan and Shaw, 1994). The component-wise organization of FAST affords access to an infinite variety of customizable queries and workflows on biological sequence data using a small command vocabulary and combinatorial logic. Component-based software is easier to learn, maintain and extend. It also makes it easy for users to interactively develop new protocols through the modular extension and recombination of existing protocols. As shown from the examples below, non-trivial computations may be expressed on a single line of the printed page. Thus, FAST can help empower non-biologist programmers to develop and communicate powerful and reproducible bioinformatic workflows for scientific investigations and publishing.

Open-source command-line utilities for bioinformatics such as the EMBOSS package (Rice et al., 2000), the FASTX tools (Gordon, 2009) or the scripts that come with BioPerl (Stajich et al., 2002) typically offer suites of tools with simple, well-defined functions that lend themselves to scripting, but are not necessarily designed according to the Unix toolbox philosophy specifically to interoperate through serial composition over pipes. Similarly, FaBox (Villesen, 2007) is a free and open online server with functions that overlap with FAST tools, but is not designed for serial composition. On the other hand, the Unix toolbox model has been used before in more or less more specialized bioinformatics applications such as the popular SAMTools suite (Li et al., 2009) and in the processing of NMR data (Delaglio et al., 1995). A toolsuite called bp-utils, with a similar design philosophy and some overlapping functionality with FAST, has recently been released at http://diverge.hunter.cuny.edu/labwiki/Bioutils.

We have written extensive documentation for each FAST utility along with useful error messages following recommended practice (Seemann, 2013). FAST is free and open source; its code is freely available to anyone to re-use, verify and extend through its GitHub repository.

## 2. Design and implementation of FAST tools

### 2.1. The FAST data model

The Unix Coreutils paradigm allows users to treat plain-text files and data streams as databases in which *records* correspond to single lines containing *fields* separated by *delimiters* such as commas, tabs, or strings of white-space characters. FAST extends this paradigm to biological sequence data, allowing users to treat collections of files and streams of multi-line sequence records as databases for complex queries, transformations and analytics. FAST generalizes the GNU Coreutils model exactly because it models sequence record *descriptions* as an ordered collection of *description fields* (see below).

Another design feature of Unix tools that also characterizes the FAST tools is their ability to accept input not only from one or more files but also from what is called *standard input*, a data-stream supported by the Unix shell, and to output analogously to *standard output*. It is this facility that allows FAST tools to be serially composed in Unix *pipelines* that compactly represent an infinite variety of expressive bioinformatic workflows.

The default data exchange format for FAST tools is the universally recognized FastA format (Lipman and Pearson, 1985). While no universal standard exists for this format, for FAST, “FastA format” means what is conventionally called “multi-fasta” format of sequence or alignment data, largely as implementated in BioPerl in the module Bio::SeqIO::fasta (Stajich et al., 2002).

In the FAST implementation of FastA format, multiple sequence records may appear in a single file or input stream. Sequence data may contain gap characters. The logical elements (or fields) of a sequence record are its *identifier*, its *description* and its *sequence*. The identifier (indicated with id in the illustration below) and description (desc) together make the *identifier line* of a sequence record, which must begin with the sequence record start symbol > on a single line. The description begins after the first block of white-space on this line (indicated with <space>). The *sequence* of a record appears immediately after its identifier line and may continue over multiple lines until the next record starts.

In FAST, users may alter how description fields are defined in sequence records by using Perl-style *regular expressions* to define delimiters (indicated by <delim>). FAST uses one-based indexing of description fields.

The FAST data model is illustrated as follows:

>seq1-id<space>seq1-desc-field1
 <delim>seq1-desc-field2<delim>...
seq1-sequence
seq1-sequence
...
seq1-sequence
>seq2-id<space>seq2-desc-field1
 <delim>seq2-desc-field2<delim>...
seq2-sequence
seq2-sequence
...
seq2-sequence

In FAST, the sequence identifier is thought of as the 0th field of the identifier line. One-based indexing of description fields in FAST is therefore consistent with zero-based indexing in Perl and one-based indexing of sequence coordinates, making all indexing consistent and uniform in FAST.

Most FAST tools extend the field-based paradigm further by supporting *tagged values* in sequence record descriptions. Tagged values are name-value pairs with a format “name=value" as common in General Feature Format (GFF) used in sequence annotation (see e.g., https://www.sanger.ac.uk/resources/software/gff/) or an alternative “name:value” format that certain FAST tools themselves can annotate in-line into sequence records by appending a new field to sequence record descriptions. Support for tagged values in FAST makes it possible to operate on sequence records with unordered or heterogeneous description fields.

### 2.2. Overview of the FAST tools

FAST utilities may be assigned to categories according to their default behavior and intended use. There are FAST tools for selection of data from sequence records, transformation of data, annotation of sequence record descriptions with computed characteristics of the data, and analysis. A complete description of all utilities included in the first major release of FAST is shown in Table 1.

**Table 1:** **Utilities in first major release of FAST**.

**Tool/Category**	**Function**	**Coreutil analog**	**Operates by default upon**
**SELECTION**			
fasgrep	Regex selection of records	grep	Identifiers
fasfilter	Numerical selection of records		Identifiers
fastax	Taxonomic selection of records		Descriptions
fashead	Order-based selection of records	head	Records
fastail	Order-based selection of records	tail	Records
fascut	Index-based selection and reordering of data	cut	Sequences
gbfcut	Extract sequences by regex matching on features		Features
alncut	Selection of sites by content		Sites
gbfalncut	Selection of sites by features		Sites
**TRANSFORMATION**			
fassort	Numerical or text sorting of records	sort	Identifiers
fastaxsort	Taxonomic sorting of records		Identifiers
fasuniq	Remove or count redundant records	uniq	Records
faspaste	Merging of records	paste	Sequences
fastr	Character transformations on records	tr	Identifiers
fassub	Regex substitutions on records		Identifiers
fasconvert	Convert sequence formats		Records
**ANNOTATION**			
faslen	Annotate sequence lengths		Descriptions
fascomp	Annotate monomeric compositions		Descriptions
fascodon	Annotate codon usage		Descriptions
fasxl	Annotate biological translations		Descriptions
fasrc	Annotate reverse complements		Descriptions
**ANALYSIS**			
alnpi	Molecular population genetic statistics		Sites
faswc	Tally sequences and characters	wc	Sequences

**Figure 1:**
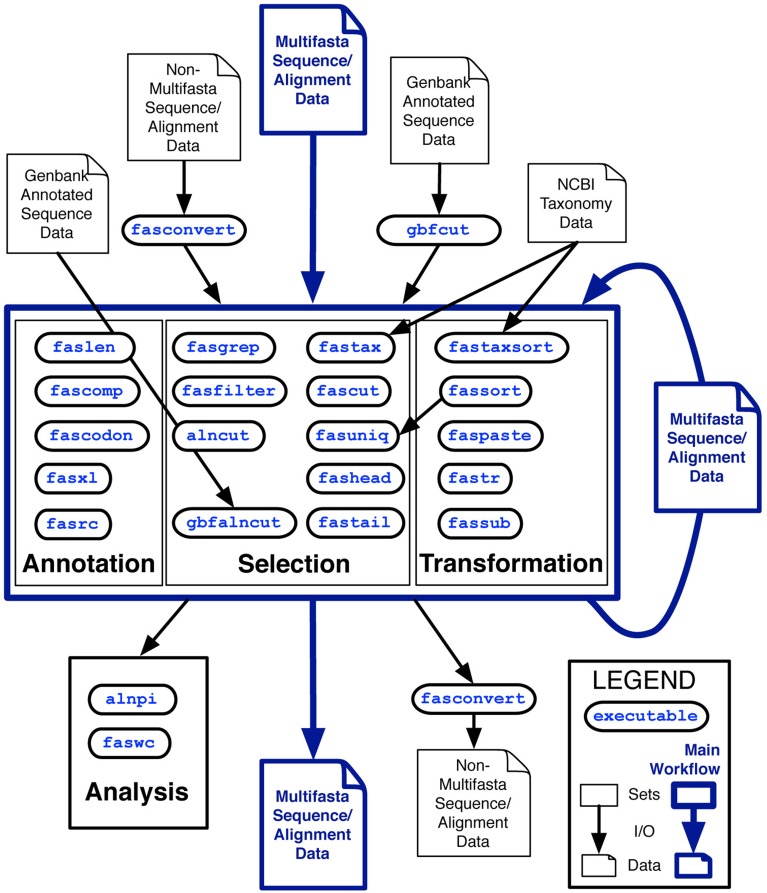
**Overview of the first major release of FAST with data and workflow dependencies indicated**. Inputs to FAST tools are shown at the top of the figure with outputs at the bottom. Outlined in blue is the primary working model, in which Multi-fastA sequence or alignment data is successively annotated, selected upon and transformed into new Multi-fastA data, or fed into a utility in the analysis category for tabular output of data summaries. Many of the utilities in the annotation category are also optionally capable of tabular output.

The analysis class is distinguished from the other classes because by default, these utilities output tables of plain-text data rather than sequence record data in FastA format. Two other tools, fasconvert and gbfcut, are designed to either input or output FastA format sequence records by default. Standardization of the FAST data model allows users to serially compose FAST tools into pipelines at the Unix command-line, which is indicated as the “main workflow” in the overview of the project shown in Figure 1.

### 2.3. General implementation and benchmarking

The BioPerl backend of FAST 1.x is version 1.6.901 downloaded in January, 2012. Bio::SeqIO components were updated to version 1.6.923 on June 4, 2014 and some Bio::Root components were updated on July 10, 2014 (github commit 50f87e9a4d). We introduced a small number of customizations to the BioPerl code-base, primarily to enable the translation of sequences containing gaps. All of the BioPerl dependencies of FAST are isolated under its own FAST name-space.

To help reduce the overall installation footprint of FAST, BioPerl dependencies of FAST scripts were analyzed with the Cava packager (http://www.cavapackager.com).

**Figure 2:**
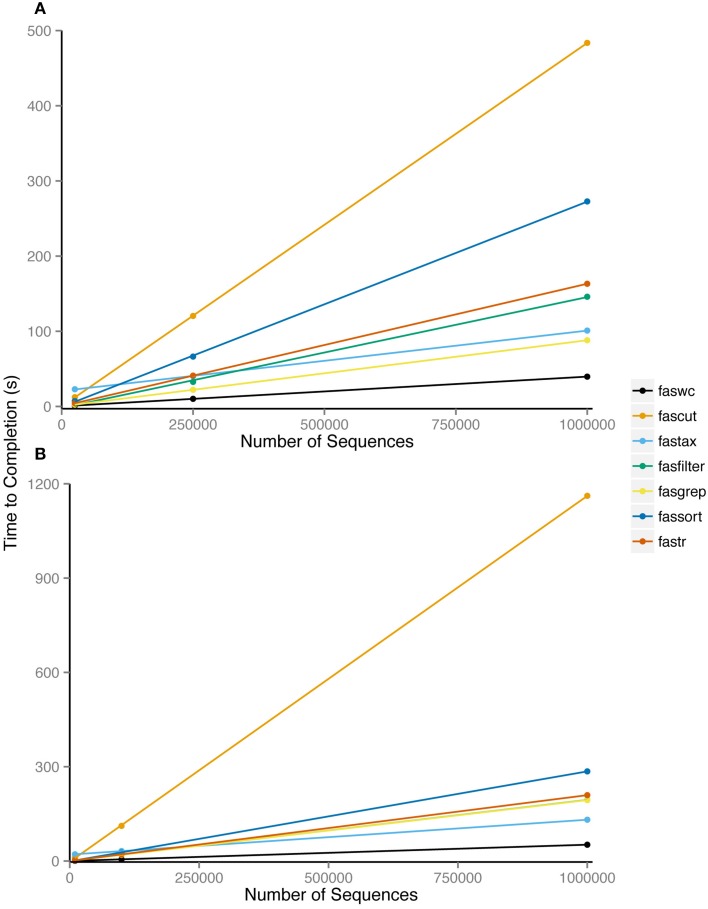
**Average processor time of 100 repetitions required to complete analysis using indicated utility**. Utilities were run on six datasets consisting of **(A)** 25,000, 2,50,000, and 10,00,000 100 bp sequences and **(B)** 10,000, 1,00,000, and 10,00,000 1000 bp sequences.

Nearly all FAST utilities process sequence records inline and therefore have linear runtime complexity in the number of sequences. Exceptions are fassort and fastail which both require some paging of data into temporary files. We performed benchmarking of FAST tools using randomly generated sequences of even composition sourced generated in Python and the Benchmark v1.15 Perl module on a MacBook Pro 2.5 Ghz Intel i7, with 8 Gb of RAM. We examined average CPU runtime over 100 replicates, comparing input sizes of 25K, 250K, or 1M sequence records of length 100, 10K, 100K, or 1M bp. Our benchmarking results show that despite data paging, fassort runtimes scale linearly with input size (Figure 2).

FAST is not designed to be fastest at computing its solutions. Rather the fastness of FAST lies in how quickly an adept user can interactively prototype, develop, and express bioinformatic workflows with it.

### 2.4. Installation and dependencies

FAST requires a working Perl installation, with official releases distributed through the Comprehensive Perl Archive Network (CPAN). A small footprint of BioPerl dependencies has been packaged together in the FAST namespace. Other CPAN dependencies may be detected and installed by the cpan package manager. A fully automated install from CPAN may on many systems be initiated by executing perl -MCPAN -e 'install FAST'. A manual install follows standard Perl install procedure. After downloading and unpacking the source directory, change into that directory and execute: perl Makefile.PL; make; make test; (sudo) make install.

We recommend that first-time users first complete the automated install from CPAN which will handle prerequisites, and then download and open the source code directory in order to practice the example usage commands (such as those in the sequel) on sample data provided within.

### 2.5. Implementation and usage of individual tools

Further implementation and usage details of individual FAST tools follows. Usage examples for individual tools refer to example data that ships with the FAST source-code installer, available from CPAN. The most recent version at the time of publication is 1.06, available from http://search.cpan.org/~dhard/FAST-1.06/ and as Supplementary Material. However we recommend to use the most recent version of FAST. For maximum reproducibility, always cite the version number when publishing results with FAST. These usage examples should be able to run from within the installation directory after installation has completed.

**fasgrep** supports *regular expression*-based selection of sequence records. FAST uses Perl-style regular expressions, which are documented freely online and within Perl, and are closely related to Unix extended regular expressions. For reference on Perl regular expressions, try executing man perlre or perldoc perlre. For example, to print only protein sequences that do *not* start with M for methionine, execute:
fasgrep -s -v ″^M″ t/data/P450.fas
In the above command the -s option directs fasgrep to search the sequence data of each record. The -v option directs fasgrep to print records that *do not* match the pattern given by its argument, which is the regular expression ^M, in which the *anchor* ^ specifies the beginning of the sequence data. fasgrep uses the BioPerl Bio::Tools::SeqPattern library to support ambiguity expansion of IUPAC codes in its regular expression arguments. Thus, to show that a segment of *Saccharomyces cerevisiae* chromosome 1 contains at least one instance of an “Autonomous Consensus Sequence” characteristic of yeast origins of replication (Leonard and McHali, 2013), look whether the following command outputs a sequence or not (note that all commands reproduced here should be entered on a single line at the Unix shell prompt):
fasgrep -se 'WTTTAYRTTTW'
t/data/chr01.fas
which is equivalent to:
fasgrep -se '[AT]TTTA[CT][AG]TTT[AT]'
t/data/chr01.fas
These examples demonstrate queries on sequence data, but fasgrep may be directed to search against other parts of sequence records including identifiers, descriptions, fields and more.
**fasfilter** supports precise numerical-based selections of sequence records from numerical data in identifiers, descriptions, fields or tagged-values in descriptions. fasfilter supports *open ranges* such as 100-, meaning “greater than or equal to 100,” closed ranges like 1e6-5e8 (meaning 1 × 10^6^ to 5 × 10^8^) and compound ranges such as 200–400, 500-. Ranges may be specified in Perl-style (or GenBank coordinate style) like from..to, in R/Octave-style like from:to or UNIX cut-style as in from-to. For example, to print records with gi numbers between 200 and 500 million, try executing:
fasfilter -x ″gi\|(\d+)″ 2e8..5e8
t/data/P450.fas
This example uses the -x option which directs fasfilter to filter on the value within the *capture buffer* which occurs within the left-most pair of parentheses of the argument, here (\d+), and \d+ is a regular expression matching a string of one or more digits from 0 to 9. The backslash after gi in the first argument quotes the vertical bar character to make it literal, since the vertical bar character is a special character in regular expressions.
**fascut** supports index-based selections of characters and fields in sequence records allowing repetition, reordering, variable steps, and reversals. Ranges are specified otherwise similarly to fasfilter. Negative indices count backwards from last characters and fields. fascut outputs the concatenation of data selections for each sequence record. Variable step-sizes in index ranges conveniently specify first, second or third codon positions in codon sequence records, for example. Examples using this syntax appear in the sequel. To print the last ten residues of each sequence, execute:
fascut -10..-1 t/data/P450.fas
**alncut** implements content-based selection of sites in alignments including gap-free sites, non-allgap sites, variable or invariant sites and parsimoniously informative sites, or their set-complements, all with the option of state-frequency-thresholds applied per site. By default, alncut prints only invariant sites. To print the set-complement, or only variable sites, use the -v option:
alncut -v t/data/popset_32329588.fas
To print sites in which no more than two sequences contain gaps, execute:
alncut -gf 2 t/data/popset_32329588.fas
**gbfcut** allows annotation-based sequence-extraction from GenBank format sequence files, useful for extracting all sequences that correspond to sets of the same type of annotated features in genome data. For example, to output 5′ and 3′ Untranslated Region (UTR) sequences from a GenBank formatted sequence of a gene, we use the -k option to restrict matching to features whose “keys” match the regular expression “UTR”:
gbfcut -k UTR t/data/AF194338.1.gb
gbfcut can handle split features such as a coding region (CDS) that is split over several exons:
gbfcut -k CDS t/data/AF194338.1.gb
More fine-grained queries of features are possible using qualifiers defined with the -q option. Multiple qualifiers may be provided at once, specifying the selection of records for which all qualifiers apply (conjunction). For example, compare the output of the following two commands:
gbfcut -k tRNA t/data/mito-ascaris.gb
gbfcut -k tRNA -q product=Ser
-q note^AGN t/data/mito-ascaris.gb
The second command queries for features with key “tRNA” containing at least one qualifier “/product” whose value matches the string literal “Ser” and no qualifiers of type “/note” whose values match the string literal “AGN.”
**gbfalncut** automates the selection of sites from alignments that correspond to one or more features annotated on one of the sequences in a separate GenBank record. This workflow eliminates the need for manual entry of coordinates and implements a useful bioinformatic query in terms of known and reproducible quantities from public data and sequence records, allowing users to query sites based on biological vocabularies of sequence features. For an example of its use see the section “Composing Workflows in FAST” in the sequel.
**faspaste** concatenates data from records input in parallel from multiple data-streams or files, record-by-record. The user may paste data from the standard input stream and from multiple input files, in an order defined by the arguments. Records from standard input may be used multiple times in concatenating data. Like in some implementions of the Unix tool paste, a hyphen input argument - to faspaste refers to the standard input stream and may be used more than once as an input argument. For maximum configurability, faspaste concatenates only one data field type (i.e., sequences or descriptions) at a time. Users may select which data stream will provide templates to receive concatenated data in output records. For example, to paste sequences of corresponding records from two data-files together and output them with the identifiers and descriptions of the data in the first file, execute:
faspaste data1.fas data2.fas
See the sequel for more advanced usage examples with faspaste.
**fassort** and **fasuniq** are designed to be often used together in Unix pipelines. The fassort utility implements numerical and textual sorting of sequence records by specific fields. The fasuniq utility removes (and optionally counts) records that are redundant with respect to a specific field, such as sequences or identifiers. In the implementation of fassort, pages of data are sorted with optimized routines in Perl Sort::Key that, if necessary, are written to temporary files and merged with Sort::MergeSort. Like its Unix Coreutil analog uniq, fasuniq compares only immediately successive input records. Therefore, users will usually want to first sort data with fassort before passing it to fasuniq. To illustrate, the following example combines and sorts input records from two instances of the same file, and then counts and removes each redundant record:
fassort -s t/data/P450.fas
t/data/P450.fas | fasuniq -c
This example illustrates that the same file may be specified as an input stream more than once to any FAST command.
**fastax** and **fastaxsort** implement taxonomic searching and sorting of sequence records, whose records are already annotated with NCBI taxonomic identifiers using taxonomic data from NCBI taxonomy (Benson et al., 2009; Sayers et al., 2009). For example, a query of “Metazoa” would match records labeled *“Homo sapiens,” “Drosophila melanogaster,”* and “Lepidoptera” but not *“Candida albicans”* or “Alphaproteobacteria.” Taxonomic selections may be logically negated and/or restricted to only those records containing valid NCBI taxonomic identifiers. Purely for historical reasons, the internal implementation of NCBI taxonomic data is custom to FAST rather than the Bio::Taxonomy libraries in BioPerl. A sample of data from tRNAdb-CE (Abe et al., 2014), in which data records are annotated with valid NCBI taxonomic identifiers in specific description fields, is included with the FAST installation package. After downloading datafiles “nodes.dmp” and “names.dmp” from NCBI Taxonomy, the following command filters sequences from Rhizobiales, assuming that records are labeled with their species (and strain) of origin in the third field of the description of the sample data file:
fastax -f 3 -S ″ \| ″
nodes.dmp names.dmp Rhizobiales
t/data/tRNAdb-CE.sample2000.fas
**fastr** and **fassub** handle, respectively, character- and string-based transformations of sequence records. The utility fastr handles character-based transliterations, deletions and “squashing” (deletion of consecutive repeats), sequence degapping, and restriction or remapping of sequence data to strict or IUPAC ambiguity alphabets. For example, to lower-case all sequence characters, execute:
fastr -s 'A-Z' 'a-z' t/data/P450.fas
Degapping requires only the simple command:
fastr --degap t/data/P450.clustalw2.fas
The utility fassub allows more arbitrary substitutions on sets of strings matched to Perl regexes, implemented through direction of the Perl s/// substitution operator on specific fields. Capture buffers may be used to refer to matched data in substitutions, for example, to reverse the order of genus and species in a file in which scientific names occur in descriptions enclosed with square brackets:
fassub -d '\[(\w+) (\w+)\]' '[$2 $1]'
    t/data/P450.fas
**fascomp, fasxl** and **fascodon** provide for annotation and analytics of compositions, translations, and codon usage frequencies of sequence records (with start and stop codons counted distinctly, in the last case). All genetic codes included in BioPerl, ultimately from NCBI Entrez, are supported.
**alnpi** outputs molecular population genetic statistics cited in Table 2 for each alignment on input. It can output a set of statistics for each alignment on input in plain text or 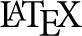 format. alnpi also supports sliding window and pairwise analysis of input data. Data and command examples are provided to reproduce the tables and sliding window analyses of statistics published in Ardell et al. (2003). Purely for historical reasons, alnpi does not use the perlymorphism routines in the BioPerl library Bio::PopGen (Stajich and Hahn, 2005). However, all of the code for these calculations has been reviewed and compared against calculations produced from DNASP (Librado and Rozas, 2009) as described previously (Ardell, 2004).

**Table 2:** **Molecular population genetic statistics in FAST**.

**Statistic**	**Symbol**	**Citation**
Number of sequences	*n*	
Number of alleles/distinct sequences	*k*	
Number of segregating sites	*S*	
Fraction of segregating sites	*s*	
Average number of pairwise differences		Nei and Li, 1979
Nucleotide diversity	π	Nei and Li, 1979
Watterson estimator	θ_*W*_	Watterson, 1975
Expected number of alleles	*E*(*K*)	Ewens, 1972
Tajima's D	*D*	Tajima, 1989
Fu and Li's D^*^	D^*^	Fu and Li, 1993
Fu and Li's F^*^	F^*^	Fu and Li, 1993; Simonsen et al., 1995
Fu and Li's Eta S	η_*S*_	Fu and Li, 1993
Fu and Li's Eta	η	Fu and Li, 1993

## 3. Composing workflows in FAST

Here we show how to interactively prototype a pipeline that computes the sliding window profile of Tajima's *D* of Figure 4A in (Ardell et al., 2003) from a publicly available datafile. The datafile associated to this figure is an NCBI PopSet with accession ID 32329588 containing an alignment of a fully annotated ciliate gene (accession AF194338.1) against several partially sequenced allelic variants. One of the variants with accession ID AY243496.1 appears to be partly non-functionalized.

First to see this data, we view it in the pager less (press “q” to quit and “space” to page):

less t/data/popset_32329588.fas

A key feature of the Unix shell allows users to recall previous commands in their so-called *history*, usually by typing the “up-arrow,” for possble re-use and editing. To check the number of sequences and characters in the alignment, execute:

faswc t/data/popset_32329588.fas

To compute our population genetic statistics we wish to remove the annotated reference sequence, the deactivated allele, and one additional sequence from analysis, which we can do using fasgrep, and verify that it reduced data by the correct number of records (six) by piping to faswc (the command is broken over two lines here but may be entered as one line on the Unix prompt):

fasgrep -v ″(AF194|349[06])″
  t/data/popset_32329588.fas | faswc

We can check the identifier lines by modifying the end of this pipeline:

fasgrep -v ″(AF194|349[06])″
  t/data/popset_32329588.fas | grep \>

Sequencing ambiguities and gap-characters can introduce noise and uncertainty in the execution and documention of bioinformatic workflows. For some computations, for example in molecular population genetics, one may want to be conservative and remove ambiguity- and gap-containing sites from an alignment. We can check for ambiguities in our data by outputing a composition table:

fasgrep -v ″(AF194|349[06])″
   t/data/popset_32329588.fas | fascomp
     --table

To remap ambiguities to gap characters, with the intent of removing all sites containing either ambiguities or gaps, we may use fastr to remap all non-strict DNA characters to gap (–) and verify the result using fascomp again:


fasgrep -v ″(AF194|349[06])″
t/data/popset_32329588.fas |
  fastr --strict -N - | fascomp --table

Now, with confidence in our remapping, we extract exclusively gap-free sites from the alignment using alncut, and verify that we reduced alignment size with faswc:

fasgrep -v ″(AF194|349[06])″
 t/data/popset_32329588.fas |
 fastr --strict -N - | alncut -g | faswc

Finally, we pass the verified pipeline output to alnpi for sliding-window analysis of Tajima's *D* in overlapping windows of width 100 and step size 25:

fasgrep -v ″(AF194|349[06])″
t/data/popset_32329588.fas |
fastr --strict -N - | alncut -g | alnpi
--window 100:25:d

## 4. Further FAST workflow examples

### 4.1. Selecting sites from alignments by annotated features

Another example, that reproduces a published result from (Ardell et al., 2003), demonstrates the utility of combining gbfalncut with alnpi, allowing users to select sites from alignments corresponding to features annotated on one of the sequences in a separate GenBank file. For example, to calculate a Tajima's *D* statistic for 5′ UTRs, corresponding to the the last line in Table 1 of that work, execute:

gbfalncut -k t/data/AF194338.1.gb 5.UTR
t/data/popset_32329588.fas | fasgrep -v
 ″(AF194|349[06])″ | fastr --strict -N
 - | alncut -g | alnpi

### 4.2. Selecting sequences by encoded motifs

An advantage of the annotation approach in FAST is the ability to select and sort sequences by attributes computed and annotated into data by utilities upstream in the pipeline. For example, to select protein-coding genes from a file cds.fas whose translations contain the *N*-glycosylation amino acid motif (Kornfeld and Kornfeld, 1985), one could execute:


fasxl -a cds.fas | fasgrep -t xl0
 ″N[^P][ST][^P]″ | fascut -f 1..-2

The first command in the pipeline translates each sequence and appends the translation to the description with the tag “xl0” (indicating translation in the zeroth reading frame). The second command in the pipeline uses a regular expression to represent the *N*-glycosylation amino acid motif pattern as the value of a “name:value” pair in the description with tag “xl0,” hence processing the annotations produced by fasxl. The regex argument to fasgrep is quoted to protect the argument from interpretation by the shell. The last command in the pipeline removes the last field in the description, restoring records as they were before they were annotated by fasxl.

### 4.3. Sorting records by third codon position composition

Another example illustrates the powerful expression of ranges in fascut. An optional “by” parameter in ranges allows increments or decrements in steps larger than one. To extract third-position bases from codon sequence records, compute and annotate their compositions into record descriptions, ultimately sorting records by their third-position adenosine contents, do:

fascut 3:-1:3 cds.fas | fascomp | fassort
 -nt comp_A

### 4.4. More advanced merging of data records

More advanced usage of faspaste requires Unix pipelines. For example to join **both** descriptions and sequences from two data-files, execute:

faspaste data1.fas data2.fas | faspaste
 -d - data2.fas

The hyphen second argument (-) to the second instance of faspaste refers to the input received from standard input through the pipe. This example works because by default, faspaste uses (“mutates”) records from the data stream named in its first argument to receive the data concatenated from all records.

To prepend the first sequence of one file repeatedly to every sequence in another file, execute:


fashead -n 1 t/data/fasxl_test4.fas |
 faspaste -r - t/data/fasxl_test4.fas

To prepend the first sequence of one file repeatedly to every other sequence in another file, using identifiers and descriptions from the second file in the output, execute:


fashead -n 1 t/data/fasxl_test3.fas |
 faspaste -r -R 2 - t/data/fasxl_test4.fas

## 5. Further documentation and usage examples

Upon installation, FAST generates and installs a complete man page for each FAST utility, which should be accessible by one or both of the following commands:

man fasgrep
perldoc fasgrep

In addition, a FAST Cookbook has been contributed by the authors and is available with the source code distribution or from the project GitHub repository at https://github.com/tlawrence3/FAST.

## 6. Concluding remarks and future directions

Planned additions in future versions of FAST include fasrand and alnrand for automated sampling, permutations and bootstrapping of sequences and sites, respectively, and fasgo and fasgosort for selection and sorting of records by Gene Ontology categories (The Gene Ontology Consortium, 2015).

## Availability

Stable versions of FAST are released through the Comprehensive Perl Archive Network (CPAN) at http://search.cpan.org/~dhard/. Development of FAST is through its GitHub repository at https://github.com/tlawrence3/FAST. For latest news on the FAST project please check the Ardell Lab homepage at http://compbio.ucmerced.edu/ardell/software/FAST/.

## Author contributions

DA conceived, designed, and wrote much of FAST. TL. contributed major code factorizations and reorganization and fastail. KK contributed code including faspaste, and fashead. RL contributed an analysis of code dependencies for the FAST installer. PB tested installation and running on Windows using Strawberry Perl. All authors, especially DC and CC, contributed documentation, testing, and code fixes. KA and DA wrote the FAST Cookbook. DA wrote the paper with major contributions from DC and TL All authors made minor contributions to the manuscript, reviewed the final version of the manuscript and agree to be accountable for its contents.

### Conflict of interest statement

The authors declare that the research was conducted in the absence of any commercial or financial relationships that could be construed as a potential conflict of interest.

## Supplementary material

This is an archival copy, correct at time of publication. For the most recent and up to date information, please follow the links in the main text of the article.

## References

[B1] Abe T. Inokuchi H. Yamada Y. Muto A. Iwasaki Y. Ikemura T. (2014). tRNADB-CE: tRNA gene database well-timed in the era of big sequence data. Front. Genet. 5:114. 10.3389/fgene.2014.00114 24822057 PMC4013482

[B2] Ardell D. H. (2004). SCANMS: adjusting for multiple comparisons in sliding window neutrality tests. Bioinformatics 20, 1986–1988. 10.1093/bioinformatics/bth187 15059827

[B3] Ardell D. H. Lozupone C. A. Landweber L. F. (2003). Polymorphism, recombination and alternative unscrambling in the DNA polymerase alpha gene of the ciliate *stylonychia* lemnae (alveolata; class spirotrichea). Genetics 165, 1761–1777. 14704164 10.1093/genetics/165.4.1761 PMC1462920

[B4] Baggerly K. A. Coombes K. R. (2009). Deriving chemosensitivity from cell lines: forensic bioinformatics and reproducible research in high-throughput biology. Ann. Appl. Stat. 3, 1309–1334. 10.1214/09-AOAS291

[B5] Baggerly K. A. Coombes K. R. (2011). What information should be required to support clinical omicsİ publications? Clin. Chem. 57, 688–690. 10.1373/clinchem.2010.158618 21364027

[B6] Barnes N. (2010). Publish your computer code: it is good enough. Nature 467, 753–753. 10.1038/467753a 20944687

[B7] Benson D. A. Karsch-Mizrachi I. Lipman D. J. Ostell J. Sayers E. W. (2009). GenBank. Nucleic Acids Res. 37, D26–D31. 10.1093/nar/gkn723 18940867 PMC2686462

[B8] Blankenberg D. Hillman-Jackson J. (2014). Analysis of next-generation sequencing data using Galaxy, in Stem Cell Transcriptional Networks, Vol. 1150, of Methods in Molecular Biology, ed Kidderpages B. L. (New York, NY: Springer), 21–43. 10.1007/978-1-4939-0512-6_2 24743989

[B9] Boulesteix A.-L. (2010). Over-optimism in bioinformatics research. Bioinformatics 26, 437–439. 10.1093/bioinformatics/btp648 19942585

[B10] Bradnam K. Korf I. (2012). UNIX and Perl to the Rescue!: a Field Guide for the Life Sciences (and Other Data-rich Pursuits). Cambridge: Cambridge University Press.

[B11] Casadevall A. Steen R. G. Fang F. C. (2014). Sources of error in the retracted scientific literature. FASEB J. 28, 3847–3855. 10.1096/fj.14-256735 24928194 PMC5395722

[B12] Cunningham F. Amode M. R. Barrell D. Beal K. Billis K. Brent S.. (2015). Ensembl 2015. Nucleic Acids Res. 43, D662–D669. 10.1093/nar/gku1010 25352552 PMC4383879

[B13] Delaglio F. Grzesiek S. Vuister G. W. Zhu G. Pfeifer J. Bax A. (1995). NMRPipe: a multidimensional spectral processing system based on unix pipes. J. Biomol. NMR 6, 277–293. 10.1007/BF00197809 8520220

[B14] Delescluse M. Franconville R. Joucla S. Lieury T. Pouzat C. (2012). Making neurophysiological data analysis reproducible: why and how? J. Phys. Paris 106, 159–170. 10.1016/j.jphysparis.2011.09.011 21986476

[B15] Ewens W. J. (1972). The sampling theory of selectively neutral alleles. Theor. Popul. Biol. 3, 87–112. 10.1016/0040-5809(72)90035-4 4667078

[B16] Fu Y. X. Li W. H. (1993). Statistical tests of neutrality of mutations. Genetics 133, 693–709. 8454210 10.1093/genetics/133.3.693 PMC1205353

[B17] Garlan D. Shaw M. (1994). An introduction to software architecture. Comput. Sci. Dep. 6355407

[B18] Gordon A. (2009). FASTX Toolkit. Available online at: http://cancan.cshl.edu/labmembers/gordon/fastx_toolkit/index.html [Online: accessed 25-January-2015].

[B19] Gouy M. Guindon S. Gascuel O. (2010). SeaView version 4: a multiplatform graphical user interface for sequence alignment and phylogenetic tree building. Mol. Biol. Evol. 27, 221–224. 10.1093/molbev/msp259 19854763

[B20] Huang Y. Gottardo R. (2013). Comparability and reproducibility of biomedical data. Brief. Bioinform. 14, 391–401. 10.1093/bib/bbs078 23193203 PMC3713713

[B21] Hutson S. (2010). Data handling errors spur debate over clinical trial. Nat. Med. 16:618. 10.1038/nm0610-618a 20526299

[B22] Ioannidis J. P. A. Allison D. B. Ball C. A. Coulibaly I. Cui X. Culhane A. C.. (2008). Repeatability of published microarray gene expression analyses. Nat. Genet. 41, 149–155. 10.1038/ng.295 19174838

[B23] Joppa L. N. McInerny G. Harper R. Salido L. Takeda K. O'Hara K.. (2013). Troubling trends in scientific software use. Science 340, 814–815. 10.1126/science.1231535 23687031

[B24] Knuth D. E. (1984). Literate programming. Comput. J. 27, 97–111. 10.1093/comjnl/27.2.97

[B25] Kornfeld R. Kornfeld S. (1985). Assembly of asparagine-linked oligosaccharides. Ann. Rev. Biochem. 54, 631–664. 10.1146/annurev.bi.54.070185.003215 3896128

[B26] Leonard A. C. McHali M. (2013). DNA replication origins. Cold Spring Harb. Perspect. Biol. 5:a010116. 10.1101/cshperspect.a010116 23838439 PMC3783049

[B27] Li H. Handsaker B. Wysoker A. Fennell T. Ruan J. Homer N.. (2009). The sequence alignment/map format and SAMtools. Bioinformatics 25, 2078–2079. 10.1093/bioinformatics/btp352 19505943 PMC2723002

[B28] Librado P. Rozas J. (2009). DnaSP v5: a software for comprehensive analysis of DNA polymorphism data. Bioinformatics 25, 1451–1452. 10.1093/bioinformatics/btp187 19346325

[B29] Lipman D. J. Pearson W. R. (1985). Rapid and sensitive protein similarity searches. Science 227, 1435–1441. 10.1126/science.2983426 2983426

[B30] Lushbough C. M. Jennewein D. M. Brendel V. P. (2011). The bioextract server: a web-based bioinformatic workflow platform. Nucleic Acids Res. 39 Suppl. 2, W528–W532. 10.1093/nar/gkr286 21546552 PMC3125737

[B31] Markowitz V. M. Chen I.-M. A. Palaniappan K. Chu K. Szeto E. Pillay M.. (2014). IMG 4 version of the integrated microbial genomes comparative analysis system. Nucleic Acids Res. 42, D560–D567. 10.1093/nar/gkt963 24165883 PMC3965111

[B32] McIlroy D. (1969). Mass-produced software components, in Proceedings of the 1st International Conference on Software Engineering, eds Buxton J. Naur P. Randell B. (Garmisch-Pattenkirchen), 138–155.

[B33] Morin A. Urban J. Adams P. D. Foster I. Sali A. Baker D.. (2012). Shining light into black boxes. Science 336, 159–160. 10.1126/science.1218263 22499926 PMC4203337

[B34] Nei M. Li W. H. (1979). Mathematical model for studying genetic variation in terms of restriction endonucleases. Proc. Natl. Acad. Sci. U.S.A. 76, 5269–5273. 10.1073/pnas.76.10.5269 291943 PMC413122

[B35] Oinn T. Greenwood M. Addis M. Alpdemir M. N. Ferris J. Glover K. (2006). Taverna: lessons in creating a workflow environment for the life sciences. Concurrency Comput. Pract. Exp. 18, 1067–1100. 10.1002/cpe.993

[B36] Peek J. (2001). Why Use a Command Line Instead of Windows? Available online at: http://www.linuxdevcenter.com/pub/a/linux/2001/11/15/learnunixos.html

[B37] Peng R. D. (2009). Reproducible research and biostatistics. Biostatistics 10, 405–408. 10.1093/biostatistics/kxp014 19535325

[B38] Peng R. D. (2011). Reproducible research in computational science. Science 334, 1226–1227. 10.1126/science.1213847 22144613 PMC3383002

[B39] Rampp M. Soddemann T. Lederer H. (2006). The MIGenAS integrated bioinformatics toolkit for web-based sequence analysis. Nucleic Acids Res. 34, W15–W19. 10.1093/nar/gkl254 16844980 PMC1538907

[B40] Rice P. Longden I. Bleasby A. (2000). EMBOSS: the european molecular biology open software suite. Trends Genet. 16, 276–277. 10.1016/S0168-9525(00)02024-2 10827456

[B41] Rosenbloom K. R. Armstrong J. Barber G. P. Casper J. Clawson H. Diekhans M.. (2015). The UCSC genome browser database: 2015 update. Nucleic Acids Res. 43, D670–D681. 10.1093/nar/gku1177 25428374 PMC4383971

[B42] Sayers E. W. Barrett T. Benson D. A. Bryant S. H. Canese K. Chetvernin V.. (2009). Database resources of the national Center for biotechnology information. Nucleic Acids Res. 37, D5–D15. 10.1093/nar/gkp382 18940862 PMC2686545

[B43] Seemann T. (2013). Ten recommendations for creating usable bioinformatics command line software. Gigascience 2:15. 10.1186/2047-217X-2-15 24225083 PMC4076505

[B44] Simonsen K. L. Churchill G. A. Aquadro C. F. (1995). Properties of statistical tests of neutrality for DNA polymorphism data. Genetics 141, 413–429. 8536987 10.1093/genetics/141.1.413 PMC1206737

[B45] Smith S. W. Overbeek R. Woese C. R. Gilbert W. Gillevet P. M. (1994). The genetic data environment an expandable GUI for multiple sequence analysis. Comput. Appl. Biosci. 10, 671–675. 10.1093/bioinformatics/10.6.671 7704666

[B46] Stajich J. E. Block D. Boulez K. Brenner S. E. Chervitz S. A. Dagdigian C.. (2002). The bioperl toolkit: perl modules for the life sciences. Genome Res. 12, 1611–1618. 10.1101/gr.361602 12368254 PMC187536

[B47] Stajich J. E. Hahn M. W. (2005). Disentangling the effects of demography and selection in human history. Mol. Biol. Evol. 22, 63–73. 10.1093/molbev/msh252 15356276

[B48] Stothard P. (2000). The sequence manipulation suite: JavaScript programs for analyzing and formatting protein and DNA sequences. Biotechniques 28, 1102, 1104. 10868275 10.2144/00286ir01

[B49] Stutz M. (2000). Linux and the Tools Philosophy. Available online at: http://www.linuxdevcenter.com/pub/a/linux/2000/07/25/LivingLinux.html

[B50] Tajima F. (1989). Statistical method for testing the neutral mutation hypothesis by DNA polymorphism. Genetics 123, 585–595. 2513255 10.1093/genetics/123.3.585 PMC1203831

[B51] The Gene Ontology Consortium. (2015). Gene ontology consortium: going forward. Nucleic Acids Res. 43, D1049–D1056. 10.1093/nar/gku1179 25428369 PMC4383973

[B52] Villesen P. (2007). FaBox: an online toolbox for fasta sequences. Mol. Ecol. Notes 7, 965–968. 10.1111/j.1471-8286.2007.01821.x

[B53] Waterhouse A. M. Procter J. B. Martin D. M. A. Clamp M. Barton G. J. (2009). Jalview version 2–a multiple sequence alignment editor and analysis workbench. Bioinformatics 25, 1189–1191. 10.1093/bioinformatics/btp033 19151095 PMC2672624

[B54] Watterson G. (1975). On the number of segregating sites in genetical models without recombination. Theor. Popul. Biol. 7, 256–276. 10.1016/0040-5809(75)90020-9 1145509

[B55] Wilson G. (2014). Software carpentry: lessons learned. F1000Res. 3:62. 10.12688/f1000research.3-62.v1 24715981 PMC3976103

[B56] Yates A. Beal K. Keenan S. McLaren W. Pignatelli M. Ritchie G.. (2015). The Ensembl REST API: ensembl data for any language. Bioinformatics 31, 143–145. 10.1093/bioinformatics/btu613 25236461 PMC4271150

