# Supplementary material for: FAST: FAST Analysis of Sequences Toolbox
Source: Front Genet. 2015 May 19;6:172. doi: 10.3389/fgene.2015.00172 (PMC4437040; doi:10.3389/fgene.2015.00172)
Supplement: Supplementary file 1 [file DataSheet1.ZIP › FAST-1.06/doc/FAST_Cookbook.pdf]

# FAST: Fast Analysis of Sequences Toolbox Cookbook

Katherine C.H. Amrine & David H. Ardell

April 8, 2015

## Contents

**1 Tutorials** **2**
1.1 Prelude . . . 2
1.1.1 The FAST definition of "FastA format" . . . 2
1.1.2 Use `man` pages for full documentation . . . 2
1.2 Example 1: Prototyping a pipeline to cut, reverse complement, and translate a gene by coordinate from a genome . . . 3
1.2.1 Calculating sequence length . . . 3
1.2.2 Cut out a subsequence by coordinate with `fascut` . . . 3
1.2.3 Computing reverse complement of a sequence with `fasrc` 3
1.2.4 Translating a sequence with `fasxl` . . . 4
1.2.5 Computing codon usage with `fascodon` . . . 4
1.2.6 Computing base composition with `fascomp` . . . 4
1.3 Example 2: Reformatting, selecting and transforming alignments in FAST . . . 4
1.3.1 Reformatting alignment data with `fasconvert` . . . 4
1.3.2 Selecting sequences with `fasgrep` . . . 5
1.3.3 Reformatting gap characters with `fastr` . . . 5
1.3.4 Degapping sites with `alncut` . . . 6
1.4 Example 3: partitioning files based on NCBI taxonomy . . . 6
1.4.1 Reformat the description to allow for fastax sorting . . 7
1.5 Example 4: Retrieve a subset of sequences based on a list of identifiers . . . 7
1.5.1 Note on searching for identifiers . . . 8

# 1 Tutorials

## 1.1 Prelude

### 1.1.1 The FAST definition of "FastA format"

FastA format began with the FastA search utilities of William Pearson. For FAST, "fasta format" means what is conventionally called "multi-fasta" format of sequence or alignment data, largely as implementated in BioPerl in the module `Bio::SeqIO::fasta`.

In the FAST implementation of "fasta format", multiple sequence records may appear in a single file or input stream. Sequence data may contain gap characters. The logical elements of a sequence record are its *identifier*, *description* and *sequence*. The *identifier* (indicated with `id` in the example here) and *description* (`desc`) together make the *identifier line* of a sequence record, that must begin with the sequence record start symbol `>` on a single line. The *description* begins after the first block of white-space on this line (indicated with `<space>`). The *sequence* of a record appears immediately after its identifier line and may continue over multiple lines until the next record.

In FAST, the description may be broken into multiple *fields* defined by a *delimiter* (indicated with `<delim>`). FAST uses a "one-based" indexing of fields as indicated here:

```
>seq1-id<space>seq1-desc-field1<delim>seq1-desc-field2<delim>...
seq1-sequence
seq1-sequence
...
seq1-sequence
>seq2-id<space>seq2-desc-field1<delim>seq2-desc-field2<delim>...
seq2-sequence
seq2-sequence
...
seq2-sequence
```

### 1.1.2 Use `man` pages for full documentation

All FAST utilities follow UNIX conventions in having default and optional behaviors. For more information about how to use and modify the behavior of any FAST utility such as `faswc`, consult its manual page with *e.g.*:

```
man faswc
```

or alternatively:

```
perldoc faswc
```

## 1.2 Example 1: Prototyping a pipeline to cut, reverse complement, and translate a gene by coordinate from a genome

### 1.2.1 Calculating sequence length

Chromosome 1 from the *Saccharomyces cerevisiae* genome is available in `t/data/chr01.fsa`. By default, `faslen` calculates the lengths of sequence records on its input, and outputs its input, augmenting sequence descriptions with its calculations using the tag (or name) `length` and a (name,value) separator `:`, as in `length:872`. We can therefore easily obtain the length of this chromosome sequence as follows:

```
faslen t/data/chr01.fsa | egrep ">"
```

Alternatively, `faswc -c` will output the length of the chromosome directly to `STDOUT`:

```
faswc -c t/data/chr01.fsa
```

### 1.2.2 Cut out a subsequence by coordinate with `fascut`

`fascut` will cut a subsequence by coordinate. For example, suppose we know that the location of gene `YAR030C` in yeast chromosome 1 begins 186512 and ends 186853 on the minus strand. Let's cut this from our chromosome. The following code will extract this subsequence in fasta format to `STDOUT`:

```
fascut 186512..186853 t/data/chr01.fsa
```

### 1.2.3 Computing reverse complement of a sequence with `fasrc`

Knowing that this is on the minus strand, we need to obtain the reverse complement of this sequence. `fasrc` will compute this. The following code will take the output of `fascut` as its input and return the reverse complemeht in fasta file to `STDOUT`:

```
fascut 186512..186853 t/data/chr01.fsa | fasrc
```

### 1.2.4 Translating a sequence with `fasxl`

To translate this sequence, we extend the pipeline with the `fasxl` utility:

```
fascut 186512..186853 t/data/chr01.fsa | fasrc | fasxl
```

Examine the output, we will see that the peptide starts with a methionine, and ends with a stop codon, indicated by the `*` character by default.

### 1.2.5 Computing codon usage with `fascodon`

If we are interested in the codon usage of our gene, we can edit the last command-line (by typing `up-arrow` on most UNIX shells) and replace `fasxl` with `fascodon` at the end of our pipeline. `fascodon` reprints the input sequence with the counts of each codon with information on starts and stops appended to the identifier. With the following code, we can see that the most frequently used codon in this example is `AAT` (encoding an Arginine)

```
fascut 186512..186853 t/data/chr01.fsa | fasrc | fascodon
```

Appending the `-t` option to the command will give the codon frequencies for both the input sequence and the reverse complement in a verbose table format as follows

```
fascut 186512..186853 t/data/chr01.fsa | fasrc | fascodon -t
```

### 1.2.6 Computing base composition with `fascomp`

`fascomp` will return the base/protein composition of a sequence as an addition to the identifier. If we are interested in the normalized (option `-n`) base composition of the first chromosome in a clean table format (option `-t`), we can run the following:

```
fascomp -nt t/data/chr01.fsa
```

## 1.3 Example 2: Reformatting, selecting and transforming alignments in FAST

### 1.3.1 Reformatting alignment data with `fasconvert`

A file with protein sequences that match a search for "P450" is available in `t/data/P450.fas` under the FAST installation directory. Another file contains this data aligned using `clustalw` with the name `P450.clustalw2.aln`.

The `fasconvert` tool can convert from fasta to many formats, or from many formats to fasta, including clustalw to fasta as showin in the following example

```
fasconvert -i clustalw -f t/data/P450.clustalw2.aln
```

The previous command automatically saves its output to an output file of the same basename and an extension of `.fas` in the same directory of the original file. The `faswc` utility will count the total number of sequences and total number of nucleotides in a fasta file To look at the length of all sequences, use the following code.

```
faswc t/data/P450.clustalw2.fas
```

which outputs

```
9 5013 t/data/P450.clustalw2.fas
9 5013 total
```

to `STDOUT`.

### 1.3.2 Selecting sequences with `fasgrep`

We can subset the output in many ways to get information we are interested in, for example, if we want to get the original sequence with the gi number "86475799", we can use `fasgrep`, which will pull out sequences that match a Perl regular expression. By default, `fasgrep` attempts to match sequence identifiers:

```
fasgrep "86475799" t/data/P450.fas
```

We can retrieve the aligned version of this sequence as it has the same identifier

```
fasgrep "86475799" t/data/P450.clustalw2.fas
```

### 1.3.3 Reformatting gap characters with `fastr`

`fastr` may be useful when we must change specific characters based on the requirements of a bioinformatic program. For example, to reformat gap characters in a fasta-format alignment from "-" to ".".

```
fastr -s "-" "." t/data/P450.clustalw2.fas
```

### 1.3.4 Degapping sites with `alncut`

`alncut` also allows for editing of alignments based on their gap profile. This utility is useful in many applications, including selecting gap-free sites for input into phylogenetic softwares. To remove sites with at least one gap in all sequences, we can do the following:

```
alncut -g t/data/P450.clustalw2.fas
```

We can then determine the length of the alignment by executing:

```
alncut -g t/data/P450.clustalw2.fas | faslen | head -1
```

And if we are interested in retaining only unique sequences, *fasuniq* appended to the output will collapse duplicate sequences to one, appending all of the identifiers to one large identifier.

```
alncut -g t/data/P450.clustalw2.fas | faslen | fasuniq
```

## 1.4 Example 3: partitioning files based on NCBI taxonomy

The `fastax` tool is a powerful tool when one wants to partition data based on their taxonomic affiliations. We can partition large datasets for subset analyses, statistical comparisons, and other applications and preparation of data. `fastax` depends on the user supplying a file with the tree structure already defined. In this example, we will use NCBI taxonomy. The files necessary include a nodes file (in this case *nodes.dmp*) and a names file (in this case, *names.dmp*). The nodes file consists of a line for each taxonomic entry in NCBI with information about its class (superfamily, genus, etc) and its parent node, indexed by its numeric identifier. These files were retrieved from NCBI in a zipped package via FTP located at pub/taxonomy/taxdump.tar.gz. The names.dmp file will link the numeric identifier to any specific name that the entry can be named including its scientific name, common name, and alternative spellings accepted by NCBI. We will not create our own nodes and names files, but note that it can be done if the user disagrees with the NCBI taxonomic structure, or requires more specific partitions of their data.

The importance of structured sequence tags comes into play in this example. As described above, the line above the sequence in a fasta file is indexed by a ">" character followed by the identifier, followed by a space, and then everything else is located in the description. `fastax` will need more structure

around the taxonomic classification. The program, by default, will search by the description field, but the descrption field will have it's own structure. Note, if our description field only contains the species, or the TaxID, then we don't need to worry about structuring the description. If it is not the only thing in our description, then we need to modify the description a bit, or determine if there is a delimiter that already exists between the TaxID and the other components of the description.

### 1.4.1 Reformat the description to allow for fastax sorting

If we look at our P450.fas file, we see that the description consists of "P450" and then a space, and then a square bracket "[", the species name, and then a closing square bracket "]". There is currently no identifier that is unique surrounding only the species name. Open bracket and closed bracket are two different characters, and using the open bracket as a delimiter will give you the species name and the closed bracket in the second field, and this will not match correctly. We will first change our description field to one delimiter using the handy `fastr` tool. Arbitrarily, we will chose the double quote character for our description delimiter.

```
fastr -d "[]" "\"" t/data/P450.fas
```

Now in standard output, we will see fasta file-formatted text with the species name in the description in the sequence tag surrounded by quotes. In this file, the beginning of the description is in field one, and the species in field two. We can use this information to construct a command to pull out the sequences that are in the taxonomic "Pooideae" tribe. Assuming that nodes.dmp and names.dmp are in the same working directory, we can run the following.

```
fastr -d "[]" "\"" t/data/P450.fas |
      fastax -S \" -f 2 t/data/nodes.dmp t/data/names.dmp "Pooideae"
```

The output of this pipeline should be five sequences, including P450 sequences from the *Triticum aestivum* and *Lolium rigidum* species (classified as species belonging to the *Pooideae* tribe).

## 1.5 Example 4: Retrieve a subset of sequences based on a list of identifiers

`fasgrep` is a useful tool for retrieving subsets of sequences from large fasta files. Often fasta files will contain an identifier line, and then one line following with a sequence corresponding to the previous identifier. If this is

always the case, parsing fasta files is fairly simple. When the sequence that follows the identifier exists on multiple lines, the task of subsetting sequences becomes more challenging. If we have a list of sequence identifiers in the file *ids.txt*, we can write a bash wrapper, incorporating the unix `cat` command, for the fasgrep command to search for this subset of sequences as follows:

```
for i in $(cat ids.txt); do fasgrep $i sequences.fas; done > subset.fas
```

Now we have a subest of sequences located in *subset.fas* that correspond to the identifiers listed in *ids.txt*.

### 1.5.1 Note on searching for identifiers

Fasgrep works with perl regular expression syntax.It is often beneficial to code the identifiers in *ids.txt* with some sort of a line or a word anchor. For example, if you are looking for a complete identifier like `sequence_1` but have sequences in your file named `sequence_11` and `sequence_12`,

```
fasgrep "sequence_1" sequences.fas
```

will return all three sequences. If you'd like to just look for `sequence_1`, adding a line anchor, such as:

```
fasgrep "sequence_1$" sequences.fas
```

will return `sequence_1` and skip `sequence_11` and `sequence_12`. This only works if `sequence_1` is at the end of your identifier. If you are looking for `sequence_1` within a larger identifier, you may need to take extra precautions to not retrieve other names that are more specific to the structure of your identifiers.
